# Analytic real algebras

**DOI:** 10.1186/s40064-016-3334-7

**Published:** 2016-09-29

**Authors:** Young Joo Seo, Young Hee Kim

**Affiliations:** 1 Department of Mathematics, Research Institute for Natural Sci., Hanyang University, Seoul, 04763 Korea; 2 Department of Mathematics, Chungbuk National University, Cheongju, 28644 Korea

**Keywords:** Analytic real algebra, Trace, *d*-algebra, *BCK*-algebra, 26A09, 06F35

## Abstract

In this paper we construct some real algebras by using elementary functions, and discuss some relations between several axioms and its related conditions for such functions. We obtain some conditions for real-valued functions to be a (edge) *d*-algebra.

## Background

The notions of *BCK*-algebras and *BCI*-algebras were introduced by Iséki and Iséki and Tanaka (1980, 1978). The class of *BCK*-algebras is a proper subclass of the class of *BCI*-algebras. We refer useful textbooks for *BCK*-algebras and *BCI*-algebras (Lorgulescu 2008); Meng and Jun (1994); Yisheng (2006). The notion of *d*-algebras which is another useful generalization of *BCK*-algebras was introduced by Neggers and Kim (1999), and some relations between *d*-algebras and *BCK*-algebras as well as several other relations between *d*-algebras and oriented digraphs were investigated. Several aspects on *d*-algebras were studied (Allen et al. 2007; Han et al. 2010; Kim et al. 2012; Lee and Kim 1999; Neggers et al. 1999, 2000). Simply *d*-algebras can be obtained by deleting two identities as a generalization of *BCK*-algebras, but it gives more wide ranges of research areas in algebraic structures. Allen et al. (2007) developed a theory of companion *d*-algebras in sufficient detail to demonstrate considerable parallelism with the theory of *BCK*-algebras as well as obtaining a collection of results of a novel type. Han et al. (2010) defined several special varieties of *d*-algebras, such as strong *d*-algebras, (weakly) selective *d*-algebras and pre-*d*-algebras, and they showed that the squared algebra $(X,\square , 0)$ of a pre-*d*-algebra $(X, *, 0)$ is a strong *d*-algebra if and only if $(X,*, 0)$ is strong. Allen et al. (2011) introduced the notion of deformations in *d* / *BCK*-algebras. Using such deformations, *d*-algebras were constructed from *BCK*-algebras. Kim et al. (2012) studied properties of *d*-units in *d*-algebras, and they showed that the *d*-unit is the greatest element in bounded *BCK*-algebras, and it is equivalent to the greatest element in bounded commutative *BCK*-algebras. They obtained several properties related with the notions of weakly associativity, *d*-integral domain, left injective in *d*-algebras also.

In this paper we construct some real algebras by using elementary functions, and discuss some relations between several axioms and its related conditions for such functions. We obtain some conditions for real-valued functions to be a (edge) *d*-algebra.

## Preliminaries

A *d-algebra * (Neggers and Kim 1999) is a non-empty set *X* with a constant 0 and a binary operation "$*$" satisfying the following axioms:

(I) $$x*x=0$$,
(II) $$0*x=0$$,
(III) $x*y=0$ and $y*x=0$ imply $x=y$ for all $x,y \in X$.

For brevity we also call *X* a *d-algebra*. In *X* we can define a binary relation "≤" by $x \le y$ if and only if $x*y=0$.

An algebra $(X,*, 0)$ of type (2,0) is said to be a *strong d-algebra* (Han et al. 2010) if it satisfies (I), (II) and (III$^*$) hold for all $x, y\in X$, where

- (III$$^*$$) $$x*y=y*x$$ implies $$x=y$$.

Obviously, every strong *d*-algebra is a *d*-algebra, but the converse need not be true (Han et al. 2010).

### Example 1

(Han et al. 2010) Let $\mathbf{R}$ be the set of all real numbers and $e \in \mathbf{R}$. Define $x*y :=(x-y)\cdot (x-e) + e$ for all $x,y\in \mathbf{R}$ where "$\cdot$" and "−" are the ordinary product and subtraction of real numbers. Then $x*x=e; e*x= e; x*y= y*x= e$ yields $(x-y)\cdot (x-e)=0$, $(y-x)\cdot (y-e)= 0$ and $x=y$ or $x=e=y$, i.e., $x=y$, i.e., $(\mathbf{R},*, e)$ is a *d*-algebra.

However, $$(\mathbf{R},*, e)$$ is not a strong *d*-algebra. If $$x*y = y*x \Leftrightarrow (x-y)\cdot (x-e) + e = (y-x)\cdot (y-e) + e$$$$\Leftrightarrow (x-y)\cdot (x-e) = -(x-y)\cdot (y-e) \Leftrightarrow (x-y)\cdot (x-e + y-e) =0 \Leftrightarrow (x-y)\cdot (x + y - 2e) =0$$$$\Leftrightarrow (x=y$$ or $$x+y = 2e$$), then there exist $$x=e+\alpha$$ and $$y=e-\alpha$$ such that $$x+y= 2e$$, i.e., $$x*y = y*x$$ and $$x\not = y$$. Hence, axiom (III$$^*$$) fails and thus the *d*-algebra $$(\mathbf{R},*, e)$$ is not a strong *d*-algebra.

A *BCK*-*algebra* is a *d*-algebra *X* satisfying the following additional axioms:

(IV) $$((x*y)*(x*z))*(z*y)=0$$,
(V) $$(x*(x*y))*y=0$$ for all $$x,y,z \in X$$.

### Example 2

(Neggers et al. 1999) Let $X:=\{0, 1, 2, 3, 4\}$ be a set with the following table:

**Table:** 

*	0	1	2	3	4
0	0	0	0	0	0
1	1	0	1	0	1
2	2	2	0	3	0
3	3	3	2	0	3
4	4	4	1	1	0

Then $(X, *, 0)$ is a *d*-algebra which is not a *BCK*-algebra.

Let *X* be a *d*-algebra and $x \in X$. *X* is said to be *edge* if for any $x \in X$, $x*X=\{x,0\}$. It is known that if *X* is an edge *d*-algebra, then $x*0=x$ for any $x \in X$ (Neggers et al. 1999).

## Analytic real algebras

Let $\mathbf{R}$ be the set of all real numbers and let “$*$” be a binary operation on $\mathbf{R}$. Define a map $\lambda : \mathbf{R}\times \mathbf{R}\rightarrow \mathbf{R}$. If we define $x*y:=\lambda (x, y)$ for all $x, y \in \mathbf{R}$, then we call such a groupoid $(\mathbf{R}, *)$ an *analytic real algebra*.

Given an analytic groupoid $(\mathbf{R}, *)$, we define

$$\begin{aligned} tr(*, \lambda ):= \int ^{\infty }_{-\infty } \,\,\, \lambda (x, x)\,\, dx \end{aligned}$$

We call $tr(*, \lambda )$ a *trace* of $\lambda$. Note that the trace $tr(*, \lambda )$ may or may not converge. Given an analytic groupoid $(\mathbf{R}, *)$, where $x*y:= \lambda (x, y)$, if $x*x = 0$ for all $x\in \mathbf{R}$, then $tr(*, \lambda ) =0$, but the converse need not be true in general.

### Example 3

Let $$x_0 \in \mathbf{R}$$. Define

$$\begin{aligned} \lambda (x, x)= {\left\{ \begin{array}{ll} 0 & \quad \text { if } x\not = x_0, \\ 1 & \quad \text {otherwise} \end{array}\right. } \end{aligned}$$

Then $$tr(*, \lambda ) = \int ^{\infty }_{-\infty } \,\,\, \lambda (x, x)\,\, dx = 0$$, but $$\lambda (x_0, x_0)= 1\not = 0$$, i.e., $$x_0 * x_0 \not = 0$$.

### **Proposition 4**

*Let*$(\mathbf{R}, *)$*be an analytic real algebra and let*$a, b, c\in \mathbf{R}$, *where*$x*y:= ax + by + c$*for all*$x, y\in \mathbf{R}$. *If*$|tr(*, \lambda )| < \infty$, *then*$tr(*, \lambda ) = 0$*and*$x*y = a(x-y)$*for all*$x, y\in \mathbf{R}$.

### Proof

Given $x\in \mathbf{R}$, we have $x*x = (a + b)x + c$. Since $|tr(*, \lambda )| < \infty$, we have $|\int ^{\infty }_{-\infty } [(a + b) x + c] dx | < \infty$. Now $\int ^{A}_0 [(a+ b)x + c] \,\,dx = (a+b)\frac{A^2}{2} + cA= A[\frac{a+b}{2}A + c]$ for a large number *A*, so that if $|tr(*, \lambda )|<\infty$, then $a+ b = 0$ and $c=0$, i.e., we have $x*y = a(x-y)$, and thus $x*x = 0$ for all $x\in \mathbf{R}$. $\square$

### **Theorem 5**

*Let*$a, b, c, d, e, f \in \mathbf{R}$. *Define a binary operation* "$*$" *on*$\mathbf{R}$*by*

$$\begin{aligned} x*y:= ax^2 + bxy + cy^2 + dx + ey + f \end{aligned}$$

*for all*$x, y\in \mathbf{R}$. *If *$|tr(*, \lambda )|<\infty$*and*$0*x = 0$*for all*$x\in \mathbf{R}$, *then*$x*y = ax(x-y)$*for all*$x, y\in \mathbf{R}$.

### Proof

Given $x\in \mathbf{R}$, we have $x*x = (a+ b + c)x^2 + (d+e)x + f$. Let $A:= a + b + c$, $B:= d + e$. If we assume $|tr(*, \lambda )| <\infty$, then $|\int ^{\infty }_{-\infty } (Ax^2 + Bx + f) \,dx| < \infty$. Now $\int ^{L}_{0}(Ax^2 + Bx + f)\,\,dx = \frac{A}{3}L^3 + \frac{B}{2} L^2 + fL = L(\frac{A}{3} L^2 + \frac{B}{2} +f)$ for a large number *L* so that $|tr(*, \lambda )| <\infty$ implies $A= B= f = 0$, i.e., $a + b + c = 0, d+e = 0, f=0$. It follows that

1 $$\begin{aligned} x*y = (ax -cy + d)(x-y) \end{aligned}$$

If we assume $0*x = 0$ for all $x\in \mathbf{R}$, then, by (1), we have

$$\begin{aligned} 0&= {} 0*x \\ &= {} (a0 -cx + d)(0-x) \\ &= {} cx^2 - dx, \end{aligned}$$

for all $x\in \mathbf{R}$. This shows that $c=d = 0$. Hence $x*y = ax(x-y)$ for all $x, y\in \mathbf{R}$. $\square$

### **Corollary 6**

*Let*$a, b, c, d, e, f \in \mathbf{R}$. *Define a binary operation* “$*$” *on*$\mathbf{R}$*by*

$$\begin{aligned} x*y:= ax^2 + bxy + cy^2 + dx + ey + f \end{aligned}$$

*for all*$x, y\in \mathbf{R}$. *If *$x*x = 0$*and*$0*x = 0$*for all*$x\in \mathbf{R}$, *then*$x*y = ax(x-y)$*for all*$x, y\in \mathbf{R}$.

### Proof

The condition, $x*x = 0$ for all $x\in \mathbf{R}$, implies $|tr(*, \lambda )| <\infty$. The conclusion follows from Theorem 5. $\square$

### **Proposition 7**

*Let*$a, b, c, d, e, f \in \mathbf{R}$. *Define a binary operation* “$*$” *on*$\mathbf{R}$ by

$$\begin{aligned} x*y:= ax^2 + bxy + cy^2 + dx + ey + f \end{aligned}$$

*for all*$x, y\in \mathbf{R}$. *If*$|tr(*, \lambda )|<\infty$*and the anti-symmetry law holds for* “$*$”, *then*$(ax -cy + d)^2 + (ay-cx +d)^2 > 0$*for*$x\not = y$.

### Proof

If $|tr(*, \lambda )|<\infty$, then by (1) we obtain $x*y = (ax -cy + d)(x-y)$. Assume the anti-symmetry law holds for “$*$”. Then either $x*y \not = 0$ or $y*x \not = 0$ for $x\not = y$. It follows that $(x*y)^2 >0$ or $(y*x)^2 > 0$, and hence $(x*y)^2 +(y*x)^2 > 0$. This shows that $(ax -cy + d)^2 + (ay-cx +d)^2 > 0$. $\square$

Note that in Proposition 7 it is clear that if $(ax -cy + d)^2 + (ay-cx +d)^2 > 0$ for $x\not = y$, then the anti-symmetry law holds.

### **Corollary 8**

*If we define*$x*y:= ax(x-y)$*for all*$x, y\in \mathbf{R}$*where*$a\not = 0$, *then*$(\mathbf{R}, *)$*is ad-algebra*.

### Proof

It is easy to see that $x*x = 0 = 0*x$ for all $x\in \mathbf{R}$. Assume that $x\not = y$. Since $x*y = ax(x-y) = ax^2 - axy$, by applying Proposition 7, we obtain $b= -a, c=0, d = e = f = 0$. It follows that $(ax - 0y + 0)^2 + (ay - 0x + 0)^2 = a^2x^2 + a^2y^2 = a^2(x^2 + y^2) > 0$ when $a\not = 0$. By Proposition 7, $(\mathbf{R}, *)$ is a *d*-algebra. $\square$

### **Proposition 9**

*Let*$a, b, c, d, e, f \in \mathbf{R}$. *Define a binary operation* “$*$” *on*$\mathbf{R}$*by*

$$\begin{aligned} x*y:= ax^2 + bxy + cy^2 + dx + ey + f \end{aligned}$$

*for all*$x, y\in \mathbf{R}$. *If*$|tr(*, \lambda )|<\infty$*and*$x*0= x$*for all*$x\in \mathbf{R}$, *then*$x*y = (1-cy)(x-y)$*for all*$x, y\in \mathbf{R}$.

### Proof

If $|tr(*, \lambda )|<\infty$, then by (1) we obtain $x*y = (ax - cy + d)(x-y)$ for all $x, y\in \mathbf{R}$. If we let $y:=0$, then $x = x*0 = (ax + d)x$. It follows that $ax^2 + (d-1)x = 0$ for all $x\in \mathbf{R}$. This shows that $a=0, d=1$. Hence $x*y = (1-cy)(x-y)$ for all $x, y\in \mathbf{R}$. $\square$

### **Theorem 10**

*If we define*$x*y:= (ax - cy + d)(x-y)$*for all*$x, y\in \mathbf{R}$*where*$a, c, d\in \mathbf{R}$*with*$a+ c\not = 0$, *then the anti-symmetry law holds.*

### Proof

Assume that there exist $x\not = y$ in $\mathbf{R}$ such that $x*y = 0= y*x$. Then $(ax -cy + d)(x-y) = 0$ and $(ay - cx + d)(y-x) = 0$. Since $x\not = y$, we have

2 $$\begin{aligned} ax -cy + d= 0 = ay -cx + d \end{aligned}$$

It follows that $(a+c)(x-y)=0$. Since $a+c \not = 0$, we obtain $x=y$, a contradiction. $\square$

### Remark

The analytic algebra $(\mathbf{R}, *)$, $x*y= ax(x-y)$ for all $x, y\in \mathbf{R}$, was proved to be a *d*-algebra in Corollary 8 by using Proposition 7. Since $x* y= ax(x-y) = (ax - 0y + 0)(x-y)$, we know that $a + 0 = a\not = 0$. Hence the algebra $(\mathbf{R}, *)$ can be proved by using Theorem 10 also.

Note that the analytic real algebra $(\mathbf{R}, *)$ discussed in Corollary 8 need not be an edge *d*-algebra, since $x*0 = ax(x-0)= ax^2 \not = x$.

## Analytic real algebras with functions

Let $\alpha , \beta : \mathbf{R} \rightarrow \mathbf{R}$ be real-valued functions. Define a binary operation “$*$” on $\mathbf{R}$ by

3 $$\begin{aligned} x * y := \alpha (x)x + \beta (y)y + c \end{aligned}$$

where $$c\in \mathbf{R}$$.

### **Proposition 11**

*Let*$(\mathbf{R}, * )$*be an analytic real algebra defined by* (3). *If*$x * x = 0 = 0 * x$*for all*$x\in \mathbf{R}$, *then*$x * y = 0$*for all*$x, y\in \mathbf{R}$.

### Proof

Assume that $x * x = 0$ for all $x\in \mathbf{R}$. Then

$$\begin{aligned} 0 & = {} x * x \\ &= {} \alpha (x)x + \beta (x)x + c\\ &= {} [\alpha (x) + \beta (x)]x + c \end{aligned}$$

If we let $x:= 0$, then $c=0$. If $x\not = 0$, then $\alpha (x) + \beta (x) = 0$, i.e., $\beta (x) = -\alpha (x)$ for all $x\not = 0$ in $\mathbf{R}$. It follows that

4 $$\begin{aligned} x * y = \alpha (x)x - \alpha (y)y \end{aligned}$$

Assume $0 * x = 0$ for all $x\in \mathbf{R}$. Then

$$\begin{aligned} 0 &= {} 0 * x \\ &= {} \alpha (0)0 + \beta (x)x + c \\ & = {} \beta (x)x \end{aligned}$$

It follows that $\beta (x) = 0$ for all $x\not = 0$ in $\mathbf{R}$. Hence we have $x * y = 0$ for all $x, y\in \mathbf{R}$. $\square$

### **Proposition 12**

*Let*$(\mathbf{R}, * )$*be an analytic real algebra defined by* (3). *If*$x * x = 0$*and*$x * 0 = x$*for all*$x\in \mathbf{R}$, *then*$x * y = x-y$*for all*$x, y\in \mathbf{R}$.

### Proof

If we assume $x * x = 0$ for all $x\in \mathbf{R}$, then by (4) we obtain $x * y = \alpha (x)x - \alpha (y)y$. Assume that $x * 0= x$ for all $x\in \mathbf{R}$. Then $x = x * 0 = \alpha (x)x - \alpha (0)0 = \alpha (x)x$. This shows that $\alpha (x) = 1$ for any $x\not = 0$ in $\mathbf{R}$. Hence $x * y = x-y$ for all $x, y\in \mathbf{R}$. $\square$

Let $a, b_1, b_2, c, d, e: \mathbf{R} \rightarrow \mathbf{R}$ be real-valued functions and let $f\in \mathbf{R}$. Define a binary operation “$*$” on $\mathbf{R}$ by

5 $$\begin{aligned} x * y:= a(x)x^2 + b_1(x)b_2(y)xy + c(y)y^2 + d(x)x + e(y)y + f \end{aligned}$$

for all $x, y\in \mathbf{R}$. Assume $0 * x = 0$ for all $x\in \mathbf{R}$. Then

$$\begin{aligned} 0 &= {} 0 * x \\ &= {} c(x) x^2 + e(x)x + f \\ &= {} [c(x)x + e(x)]x + f \end{aligned}$$

for all $x\in \mathbf{R}$. It follows that $f= 0$ and $c(x)x + e(x)=0$ for all $x\not = 0$ in $\mathbf{R}$. Hence $c(y)y^2 + e(y)y = 0$ for all $y\in \mathbf{R}$. Hence

6 $$\begin{aligned} x * y = a(x)x^2 + b_1(x)b_2(y)xy + d(x)x \end{aligned}$$

Assume $x * x = 0$ for all $x\in \mathbf{R}$. Then by (6) we obtain

$$\begin{aligned} 0 & = {} x * x \\ &= {} a(x)x^2 + b_1(x)b_2(x)x^2 + d(x)x \end{aligned}$$

It follows that $d(x)x = -[a(x)x^2 + b_1(x)b_2(x)x^2]$. By (6) we obtain

7 $$\begin{aligned} x * y = b_1(x)x [ b_2(y)y - b_2(x)x ] \end{aligned}$$

### **Theorem 13**

*Let*$b_1, b_2: \mathbf{R} \rightarrow \mathbf{R}$*be real-valued functions. Define a binary operation* “$*$” *on*$\mathbf{R}$*as in* (7). *If we assume*$b_2(x)x \not = b_2(y)y$*and*$b_1^2(x)x^2 + b_1^2(y)y^2 > 0$*for any*$x\not = y$*in*$\mathbf{R}$, *then*$(\mathbf{R}, * )$*is ad-algebra*.

### Proof

Assume the anti-symmetry law holds. Then it is equivalent to that if $x\not = y$ then $x * y \not = 0$ or $y * x \not = 0$, i.e., if $x\not = y$ then $(x * y)^2 + (y * x)^2 > 0$. Since $x * y$ is defined by (7), we obtain that if $x\not = y$ then

$$\begin{aligned} (b_1^2(x)x^2 + b_1^2(y)y^2)(b_2(x)x - b_2(y)y)^2 > 0 \end{aligned}$$

By assumption, we obtain that $(\mathbf{R}, *)$ is a *d*-algebra. $\square$

### Example 14

Consider $x * y : = ax(x-y)$ for all $x, y\in \mathbf{R}$. If we compare it with (7), then we have $b_1(x) = a, b_2(y)= -1$ and $b_2(x) = -1$ for all $x\in \mathbf{R}$. This shows that $b_2(x)x - b_2(y)y = (-1)x - (-1)y = y-x \not = 0$ when $x\not = y$. Moreover, $b_1^2(x)x^2 + b_1^2(y)y^2 = a^2x^2 + b_1^2(y)y^2 >0$ since $a\not = 0$. By applying Theorem 13, we see that an analytic real algebra $(\mathbf{R}, *)$ where $x * y:= ax(x-y)$, $a \not = 0$ is a *d*-algebra.

### Example 15

Consider $x * y:= x\tan 2x [e^yy - e^x x]$ for all $x, y\in \mathbf{R}$. By comparing it with (7), we obtain $b_1(x) = \tan 2x, b_2(y)= e^y$ and $b_2(x) = e^x$. If $x\not = y$, then it is easy to see that $xe^x \not = ye^y$ and $b_1^2(x)x^2 + b_1^2(y)y^2 = (\tan 2x)^2x^2 + (\tan 2y)^2y^2 > 0$ when $x\not = y$. Hence an analytic real algebra $(\mathbf{R}, * )$ where $x * y:= x\tan 2x [e^yy - e^x x]$ is a *d*-algebra by Theorem 13.

In Theorem 13, we obtained some conditions for analytic real algebras to be *d*-algebras. In addition, we construct an edge *d*-algebra from Theorem 13 as follows.

### **Theorem 16**

*If we define a binary operation* “$*$” *on*$\mathbf{R}$*by*

$$\begin{aligned} x * y:= {\left\{ \begin{array}{ll} x[1-\frac{b_1(x)}{b_1(y)}] & \quad \text { if } y\not = 0, \\ x & \quad \text {otherwise} \end{array}\right. } \end{aligned}$$

*where*$b_1(x)$*is a real-valued function such that *$b_1(y) \not = 0$*if*$y\not = 0$. *Then*$(\mathbf{R}, *)$*is an edged-algebra.*

### Proof

Define a binary operation “$*$” on $\mathbf{R}$ as in (7) with additional conditions: $b_2(x)x \not = b_2(y)y$ and $b_1^2(x)x^2 + b_1^2(y)y^2 > 0$ for any $x\not = y$ in $\mathbf{R}$. Assume $x * 0 = x$ for all $x\in \mathbf{R}$. Then

$$\begin{aligned} x &= {} x * 0 \\ &= {} b_1(x)x[b_2(0)0 - b_2(x)x] \\ &= {} -b_1(x)b_2(x)x^2 \end{aligned}$$

Combining with (7) we obtain

$$\begin{aligned} x * y &= {} b_1(x)b_2(y)xy - b_1(x)b_2(x)x^2 \\ & = {} b_1(x)b_2(y)xy + x \\ &= {} x[b_1(x)b_2(y)y + 1] \end{aligned}$$

If we let $xy\not = 0$, then

$$\begin{aligned} x * y &= {} x \left[b_1(x)(-\frac{1}{b_1(y)}) + 1\right] \\ &= {} x \left[1-\frac{b_1(x)}{b_1(y)}\right] \end{aligned}$$

If we let $x * y:= x$ when $y=0$, then $(\mathbf{R}, *)$ is an edge *d*-algebra. $\square$

### Example 17

Define a map $b_1(x):= e^{\lambda x}$ for all $x\in \mathbf{R}$. Then $x * y = x[1-\frac{e^{\lambda x}}{e^{\lambda y}}]= x(1-e^{\lambda (x-y)})$ when $y\not = 0$. If we define a binary operation “$*$” on $\mathbf{R}$ by

$$\begin{aligned} x * y:= {\left\{ \begin{array}{ll} x(1-e^{\lambda (x-y)}) &{}\text { if } y\not = 0, \\ x &{} \text {otherwise}, \end{array}\right. } \end{aligned}$$

then $(\mathbf{R}, *)$ is an edge *d*-algebra.

### **Proposition 18**

*If we define a binary operation* “$*$” *on*$\mathbf{R}$*by*

$$\begin{aligned} x * y:= {\left\{ \begin{array}{ll} x[1-\frac{b_1(x)}{b_1(y)}] &{} \text { if } y\not = 0, \\ x &{} \text {otherwise} \end{array}\right. } \end{aligned}$$

*where*$b_1(x)$*is a real-valued function such that*$b_1(y) \not = 0$*if*$y\not = 0$. *Assume that if*$x\not = y$, *then either*$b_1(x * y) = b_1(x)$*or*$b_1(x * (x * y))= b_1(y)$. *Then*

8 $$\begin{aligned} (x * (x * y))* y = 0 \end{aligned}$$

*for all*$$x, y\in \mathbf{R}$$.

### Proof

By Theorem 16, $(\mathbf{R}, *)$ is an edge *d*-algebra and hence (8) holds for $x * y =0$ or $y=0$. Assume $x * y\not = 0$ and $y\not = 0$. Then

$$\begin{aligned} x * (x * y) = x \left[1-\frac{b_1(x)}{b_1(x * y)}\right] \end{aligned}$$

It follows that

$$\begin{aligned} (x* (x *y))\star y &= {} [x * (x * y)] \left[1-\frac{b_1(x * (x * y))}{b_1(y)}\right] \\ &= {} x \left[1-\frac{b_1(x)}{b_1(x * y)}\right] \left[1-\frac{b_1(x*(x* y))}{b_1(y)}\right]\\ &= {} 0, \end{aligned}$$

proving the proposition. $\square$

## Conclusions

We constructed some algebras on the set of real numbers by using elementary functions. The notions of (edge) *d*-algebras were developed from *BCK*-algebras, and widened the range of research areas. It is useful to find linear (quadratic) polynomial real algebras by using the real functions. In "Analytic real algebras" section, we obtained some linear (quadratic) algebras related to some algebraic axioms, and found suitable binary operations for (edge) *d*-algebras. In "Analytic real algebras with functions" section, we developed the idea of analytic methods, and obtained necessary conditions for the real valued function so that the real algebra is an edge *d*-algebra. We may apply the analytic method discussed here to several algebraic structures, and it may useful for find suitable conditions to construct several algebraic structures and many examples.
